# Challenges and Prospective of Enhancing Hydatid Cyst Chemotherapy by Nanotechnology and the Future of Nanobiosensors for Diagnosis

**DOI:** 10.3390/tropicalmed8110494

**Published:** 2023-11-06

**Authors:** Soheil Sadr, Narges Lotfalizadeh, Amir Mohammad Abbasi, Nooshinmehr Soleymani, Ashkan Hajjafari, Elahe Roohbaksh Amooli Moghadam, Hassan Borji

**Affiliations:** 1 Department of Pathobiology, Faculty of Veterinary Medicine, Ferdowsi University of Mashhad, Mashhad 917794897, Iran; soheil.sadr42@gmail.com (S.S.);; 2 Department of Pathobiology, Faculty of Veterinary Medicine, Islamic Azad University, Science and Research Branch, Tehran 1477893855, Iran

**Keywords:** chemotherapy, hydatid cyst, nanotechnology, nanobiosensors, diagnose

## Abstract

Hydatid cysts have been widely recognized for decades as a common medical problem that affects millions of people. A revolution in medical treatment may be on the prospect of nanotechnology enhancing chemotherapy against hydatid cysts. An overview of nanotechnology’s impact on chemotherapeutics is presented in the current review. It discusses some of the challenges as well as some of the opportunities. The application of nanotechnology to enhance chemotherapy against hydatid cysts is what this review will explore. Nanotechnology is a critical component of delivering therapeutic agents with greater precision and efficiency and targeting hydatid cysts with better efficacy, and minimizing interference with surrounding tissue. However, there are biodistribution challenges, toxicity, and resistance problems associated with nanotherapeutics. Additionally, nanobiosensors are being investigated to enable the early diagnosis of hydatid cysts. A nanobiosensor can detect hydatid cysts by catching them early, non-invasively, rapidly, and accurately. The sensitivity and specificity of diagnostic tests can be enhanced with nanobiosensors because they take advantage of the unique properties of nanomaterials. By providing more precise and customized treatment options for hydatid cysts, nanotechnology may improve therapeutic options and strategies for diagnosing the disease. In conclusion, treatment with nanotechnology to treat hydatid cysts is potentially effective but presents many obstacles. Furthermore, nanobiosensors are being integrated into diagnostic techniques, as well as helping to diagnose patients earlier and more accurately.

## 1. Introduction

Hydatid cyst disease is an important threat to people’s well-being [1]. This parasite can live on various hosts in various parts of the world, mainly pastoral and rural [2,3]. As a result of this parasitic disease, fluid-filled cysts develop, which can become substantial in size and silently destroy the host’s tissues and structures [4]. In humans and animals, silent progression can lead to a delay in diagnosis and limited treatment options, thus exacerbating the condition when it goes undiagnosed [5].

The fibrous layer of a hydatid cyst is derived from the host’s connective tissue, forming a protective barrier against the host’s immune response [6]. This is followed by a laminated layer of chitinous fibers arranged in precise laminations, which may contribute to maintaining mechanical stability and evading immune detection [7,8,9]. The innermost germinal layer produces protoscolices, an infective agent that facilitates attachment and further infection [10,11]. A parasite’s behavior in this layered structure reveals that it can adapt to its host’s environment to survive and spread on it [12].

In recent decades, research innovations and technological advances have merged to give rise to a new field of research known as nanotechnology, in which system components are engineered and designed at the nanoscale, a measurement requiring extreme accuracy and measured in billionths of meters [13,14]. This field can transcend conventional boundaries, allowing scientists to manipulate matter fundamentally [15]. The advent of nanotechnology promises to revolutionize medicine in the next few decades by opening up a whole new world of possibilities that could drastically change treatment methods [16,17]. The potential of nanotechnology to improve chemotherapy is one of the most promising aspects of its influence in the medical field [18,19]. The traditional method of chemotherapy involves administering therapeutic agents indifferently to both diseased and healthy tissues, resulting in harmful adverse effects during treatment [20,21]. The advent of nanotechnology promises to transcend this limitation by enabling these therapeutic agents to be controlled with unprecedented precision [22,23,24]. Through nanoparticles, chemotherapy drugs can be delivered to the target area with high accuracy to avoid collateral tissue injuries [25,26,27]. With a targeted delivery method, it is expected that the performance of the chemotherapy will be increased [28,29,30]. The side effects of conventional chemotherapy will be reduced, which often cause significant health problems [31,32,33]. Nanotechnology offers a promising avenue of innovation for treating hydatid cyst disease, which poses a formidable challenge to medical professionals [34,35,36]. Researchers have been capable of using nanoparticles to design treatment strategies that target cysts precisely with unique properties while minimizing adverse effects on healthy tissues due to their use [37,38,39]. With the potential to change the therapeutic landscape, this precise and efficient drug delivery system may become a saving grace for those suffering from this parasitic condition [40,41,42,43]. Hydatid cyst disease and nanotechnology synergize to create a compelling story of hope in the face of adversity [44,45,46,47].

Cyst ruptures and anaphylactic shock can be prevented by accurately diagnosing hydatid cysts. Although the diagnosis can be challenging because the condition is similar to others and reliable imaging techniques are required, several factors contribute to the challenge [48,49]. Detecting cysts in specific locations can also be problematic, adding to the diagnostic difficulties [50,51,52]. As a result, it is imperative to overcome these challenges to ensure that the treatment can occur effectively and on time. Nanobiosensors are superior tools for detecting hydatid cysts [53]. They not only offer exceptional sensitivity and versatility when it comes to detecting specific biomarkers, but they also improve diagnostic accuracy [54]. A further advantage of these nanoscale devices is that they enable rapid and non-invasive detection, which can be especially helpful for early-stage cysts, which conventional treatments would otherwise miss [55]. Through the integration of nanobiosensors with imaging technology, this technology has the potential to revolutionize hydatid cyst diagnosis [56].

Using nanotechnology as a therapeutic approach against the hydatid cyst is the objective of this review article. A review of current treatment methods will be presented, along with nanotechnology-based solutions that might be developed to solve those challenges. As well as highlighting the prospects of these devices, the current review emphasizes the value of nanobiosensors as a tool to expand the field of hydatid cyst management by highlighting their importance in the future.

## 2. Challenges in Conventional Chemotherapy

Traditionally, hydatid cysts are treated with anthelmintic drugs such as albendazole and mebendazole, staples of conventional chemotherapy [57]. *Echinococcus granulosus* (*E. granulosus*) is inhibited from growing and reproducing when treated with these medications [58]. Even though these drugs have proven effective, they also present several limitations [59]. One of the critical problems is the necessity of prolonging treatment durations, which may last several months or even years [60]. Concerns about drug-resistant *E. granulosus* emerging among patients who cannot comply with such prolonged regimens have been expressed [61,62].

**Figure 1:**
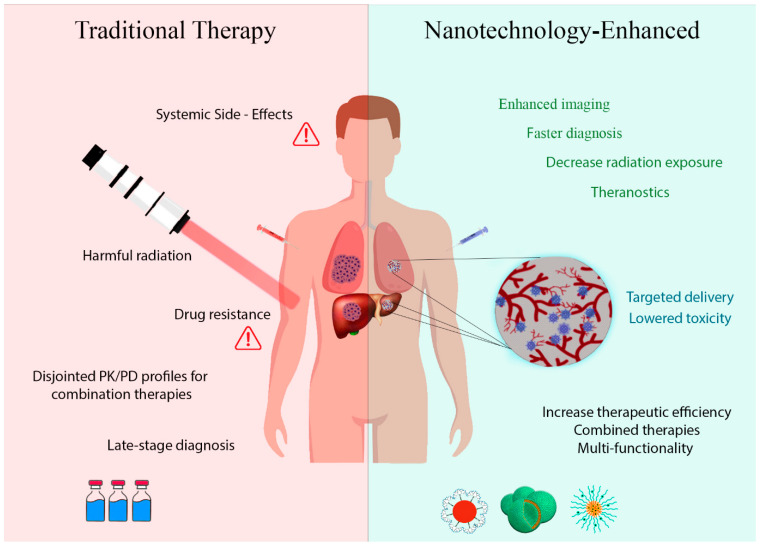
Enhancing Hydatid Cyst Therapy and Improved Diagnosis through Nanotechnology. A schematic representation illustrates nanotechnology’s dual role in managing hydatid cyst infections. Nanoparticles are employed not only to enhance chemotherapy within the cyst, but also for improved diagnostic accuracy. This integrated approach harnesses nanotechnology’s potential for precise diagnosis and targeted treatment, offering a comprehensive solution for hydatid cyst management.

Even though conventional chemotherapy is useful in treating hydatidosis, many problems still exist [63]. The procedure may need to be revised in cases of large cysts or those located in complex anatomical locations [64]. These medications have difficulty reaching parasites’ cores because cyst walls are difficult to penetrate. Long-term exposure to *E. granulosus* can result in drug-resistant strains [65,66]. These anthelmintic drugs adversely affect the patient’s quality of life and determination to continue treatment, which can cause gastroenteritis and other serious complications [67]. The intricate nature of hydatid cysts makes conventional treatment challenging since they often reside deep within key organs [68,69,70]. Therefore, it is often challenging for drugs to reach their intended targets. Considering the patients’ current health conditions and other aspects that may interfere with their health, this article suggests that treatment needs to be customized to each patient’s specific requirements (Figure 1).

## 3. Detection of Hydatid Cysts: Current Approaches and Limitations

To treat hydatid cysts effectively, it is crucial to detect them as soon as possible [71,72]. Unfortunately, it is still difficult to detect this condition promptly and reliably, given its deceptive nature and existing diagnostic limitations. However, imaging techniques, such as ultrasound, are useful for providing initial insights, but can often be misinterpreted because hydatid cysts can often be confused with other cystic lesions [73,74]. High-resolution imaging can be obtained with Computerized Tomography (CT) scans and Magnetic Resonance Imaging (MRI), though these methods are expensive and are not suitable for distinguishing cyst types [75,76,77].

Specific antibodies against *E. granulosus* antigens can be screened via serological tests, such as ELISA and Western Blotting, but these tests are also limited [78]. Unlike ELISA, the Western Blot is more specific [79,80]. However, it requires specialized equipment and proficiency, increasing the chance of false positives. The resemblance between hydatid cysts and other cystic lesions can complicate the diagnosis [81]. In addition, the current screening system often detects cysts after they have already grown considerably, thus preventing early intervention that might result in better outcomes [82].

**Figure 2:**
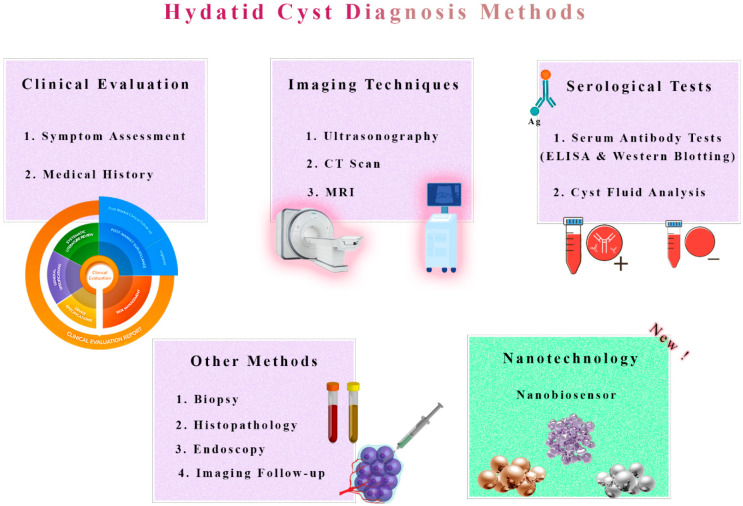
Traditional Diagnosis vs. Nanobiosensor Novelty in Hydatid Cyst Detection. A comparative representation highlights the contrast between traditional diagnostic methods and the innovative use of nanobiosensors in detecting hydatid cysts. Traditional techniques, such as imaging and serological tests, are depicted alongside the emerging nanobiosensor technology, which offers enhanced sensitivity and specificity for accurate and rapid diagnosis. This illustration underscores the potential revolution in hydatid cyst detection by nanobiosensors, promising improved early diagnosis and patient care.

Performing an aspiration and biopsy is often necessary to reach a definitive diagnosis [83]. However, these procedures are not without risk, including the possibility of rupture and cyst spreading. Sometimes, even serological tests can fail, especially when dealing with inactive cysts or cysts that have calcified over the years [84,85]. Further complicating cancer detection is the lack of advanced imaging and diagnostic technologies in areas with a low level of technology [86]. More research is needed on hydatid cysts, and more accurate and accessible diagnostic methods are required (Figure 2).

## 4. Nanotechnology and Nanoparticle Applications

Regarding biomedical progress, nanotechnology is one of the most influential breakthroughs when it is seamlessly integrated with the multifaceted scope of veterinary medicine, particularly in the specialized and complex discipline of parasitology [87]. It is no wonder that the medical field has experienced groundbreaking advances due to nanotechnology in recent years since it measures in nanometers, on a very small scale [88]. Nanotechnology offers a spectacular array of possibilities to veterinary medicine, where parasitic infections still pose significant challenges [89]. Various materials have the ability to handle the pervasive challenges of parasitic infections, each possessing distinct characteristics that contribute to their effectiveness when it comes to combating parasitic infections [90].

Metal nanoparticles, such as gold and silver nanoparticles, are well known for their outstanding stability and ability to be easily functionalized [91,92]. Parasitology organizations are becoming more adept at delivering targeted drugs to parasites [93,94]. They can attach ligands that target specific parasites or host cell receptors in addition to their surface properties. Therefore, the absorption of medications and the effectiveness of treatment is enhanced [95].

This polymeric nanoparticle made of Poly(Lactic-co-Glycolic Acid) (PLGA) and chitosan is an excellent example of a biocompatible and controlled delivery of drugs that can be used in countless applications [96,97]. Their unique contribution involves the ability to house anthelmintic drugs and release them in a controlled manner, which ensures that medicinal agents remain compelling long enough in the body to maximize therapeutic effects [98]. This sophisticated process results in a reduction in the dosing frequency and reduced side effects, achieving superior clinical outcomes [99].

Liposomes are lipid-based nanoparticles that possess malleability and biocompatibility among their most notable properties [100,101]. A versatile platform for the delivery of parasitology drugs can be developed by using these lipid ensembles [102]. Hydrophilic and hydrophobic therapeutic compounds can be encapsulated in various ways, allowing for the development of comprehensive and efficient methods for managing parasitic diseases [102].

**Figure 3:**
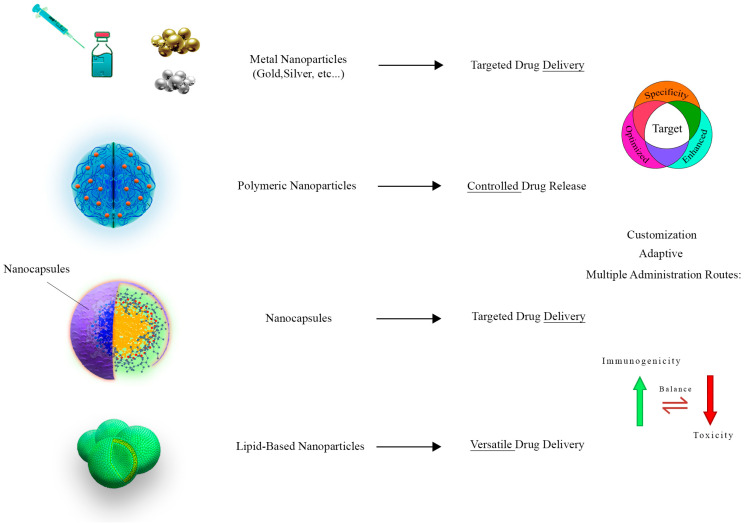
This figure shows the diverse applications of nanocarriers in the realm of parasitology. Three distinct categories of nanocarriers are featured, including metallic, polymeric, and lipid-based nanoparticles. Metallic nanoparticles, vividly exemplified by gold and silver nanoparticles, demonstrate their proficiency in targeted drug delivery, elevating treatment effectiveness by facilitating drug uptake. Polymeric nanoparticles, constructed from biocompatible materials like PLGA and chitosan, excel in controlled and sustained drug release, ultimately reducing dosing frequency and minimizing side effects. Meanwhile, lipid-based nanoparticles emerge as adaptable drug delivery platforms, adept at encapsulating hydrophilic drugs for comprehensive therapeutic approaches.

Nanocapsules are one of several nanocarriers among the wide variety of nanocarriers that serve various functions. There is a protective layer surrounding lipid or polymer capsules that contain therapeutic agents [102,103]. Utilizing this type of clever design guarantees the stability of encapsulated drugs, as well as targeted drug release, resulting in exact drug distribution right to the site of the parasite infection, providing maximum effectiveness [104]. A delicate balance must be struck to increase treatment efficacy while reducing side effects. A powerful composition for parasitic disease management is the result. Diagnostics and treatment for veterinary parasites are set to undergo a revolution as nanotechnology and veterinary parasitology merge [105,106]. Soon, as a result of recent developments in these fields, veterinary science and nanotechnology are predicted to continue to flourish, innovate, and, ultimately, redefine our approach to treating parasitic infections in companion animals (Figure 3).

## 5. Revolutionizing Chemotherapy through Nanotechnology

In the past, conventional chemotherapy has been controversial because it is non-specific, often causing adverse effects on healthy tissues and resulting in limited therapeutic effectiveness [107,108]. A new era is now underway with the advent of nanotechnology and targeted drug delivery, promising many great treatment options [109,110]. It is possible to deliver therapeutic agents precisely at disease sites through nanoparticles, which are meticulously engineered with precise dimensions and functional groups [111]. Regarding hydatid cysts, nanoparticles can be tailored to target cysts while bypassing healthy tissues [112]. A nanoparticle can harmlessly pass through the intricate barriers within the host’s body [113].

### 5.1. Metallic Nanoparticles

Various nanomaterials are used to encapsulate praziquantel, such as gold, silver, zinc, copper, and zinc oxide, all composed of a combination of metals, to deliver the drug through nano-based delivery systems [114,115,116]. Materials like these function as carriers and offer intrinsic antimicrobial properties that contribute to the therapeutic effect.

According to Napooni et al. (2019), AuNPs display noteworthy protoscolicidal abilities [117]. These gold nanoparticles can be used as an alternative treatment for cystic echinococcosis because they eliminate the side effects of chemical drugs commonly used to treat this disease. These drugs are also effective when combined with solid lipid nanoparticles that contain albendazole and praziquantel. Based on a mouse model, Jelowdar et al. (2017) found that CE’s chemoprophylaxis outperformed free albendazole and Praziquantel [118]. It is, therefore, possible to conduct further studies by conducting clinical trials with this substance.

By synthesizing silver nanoparticles derived from *Penicillium aculeatum*, Rahimi et al. (2015) displayed protoscolicidal effects against *E. granulosus* [119]. The findings showed that Ag nanoparticles at all concentrations were remarkable protoscolicidal agents. Considering their biodegradability and harmless nature, these investigators believe AgNPs could be used as protoscolicidal agents.

Ag nanoparticles may help reduce the toxic effects of albendazole, the most commonly prescribed drug for treating hydatid disease. Aside from necrosis, degeneration, and steatosis, albendazole causes elevated serum hepatic enzyme levels. Nassef et al. (2019) found that encapsulating ABZ within Ag NPs makes it more potent at eliminating cystic echinococcosis [20].

It is well documented that Ag nanoparticles, amphotericin B, hypertonic saline, and *Foeniculum vulgare* essential oil have all been demonstrated to have protoscolicidal activity by Lashkarizadeh et al. (2015) [120]. The antiparasitic activity of AgNPs has been confirmed. Protoscoleces had a maximum activity level of 4 mg/mL, and 71.6% of them had died after an hour of exposure to it.

### 5.2. Antiparasitic-Loaded Nanodrugs

Nanostructured delivery methods are becoming increasingly popular to enhance the therapeutic effect of pharmaceutical agents and improve their bioavailability [121]. There is potential for a significant improvement in clinical efficacy and bioavailability due to a revolution in medical technology [122]. Nano-based formulations of albendazole and mebendazole, two widely used anthelmintic drugs, have been found to have promising results in treating hydatid cyst disease [123,124,125].

By applying ABZ sulfoxide-loaded chitosan-PGLA NPs produced by nanoprecipitation, Darvishi et al. (2020) demonstrated their utility in vivo [35]. A comparison was conducted between hemodialysis-treated cysts and sham surgery-treated cysts, and it was found that hemodialysis showed impressive therapeutic effects. As a result, the authors concluded that the mixture of ABZ sulfoxide-loaded chitosan-PGLA nanoparticles can assist in managing hydatid cyst disease in mice. The effects of ZrO_2_ nanoparticles on *E. granulosus* protoscoleces have been examined in a similar setting by Ibrahim (2020) [126]. Upon 60 min of incubation, ZrO_2_ NPs at 1000, 2000, and 4000 g/mL exhibited a notable contribution to the death of parasites in vivo. The specific delivery of the medication at the cyst site makes it both more efficient and minimizes collateral damage, which is one of the main downsides of conventional chemotherapy. Conventional chemotherapy is less discomforting by targeting the drug directly at the cyst. A closer look at the obstacles that hinder nanotechnology’s implementation, particularly in managing hydatid cysts, is necessary to understand its promise as a therapeutic technique [127].

With nanotechnology available to assist with manufacturing drug carriers, researchers can accomplish several benefits, including controlled drug release, precisely directed drug delivery, and more excellent solubility, all of which circumvent the weaknesses of traditional drug administration methods [128,129,130]. Using albendazoles and mebendazoles nanoencapsulated is a promising method of benzimidazole anthelmintics [131]. The encapsulation of these drugs has been achieved using various nanostructured carriers, including liposomes, polymeric nanoparticles, and lipid nanoparticles [132]. This significantly improves drug stability, circulation, and sustained release. It is possible to protect albendazole and mebendazole from rapid degradation by nanoencapsulation. In addition, nanoencapsulation makes it more likely for albendazole and mebendazole to accumulate selectively at the site of action. Further, praziquantel can also be implemented into nanoformulations to combat hydatid cysts, which play a crucial role in treating helminthic infections.

There are several mechanisms by which NPs are associated with antiparasitic activity, including the induction of apoptosis [133,134,135]. In an in vitro study, Naseri et al. (2016) evaluated albendazole sulfoxide and albendazole sulfoxide-loaded poly (lactic-co-glycolic acid)-PEG against protoscoleces [136]. In terms of apoptosis, they showed that ABZs and ABZs-loaded PLGA-PEG induced the cell death of protocoleces with oligonucleosomal DNA fragmentation. As a method for assessing whether these ABZs induce apoptosis in protoscoleces, caspase-3 mRNA synthesis by the genome of *E. granulosus* was tested.

**Figure 4:**
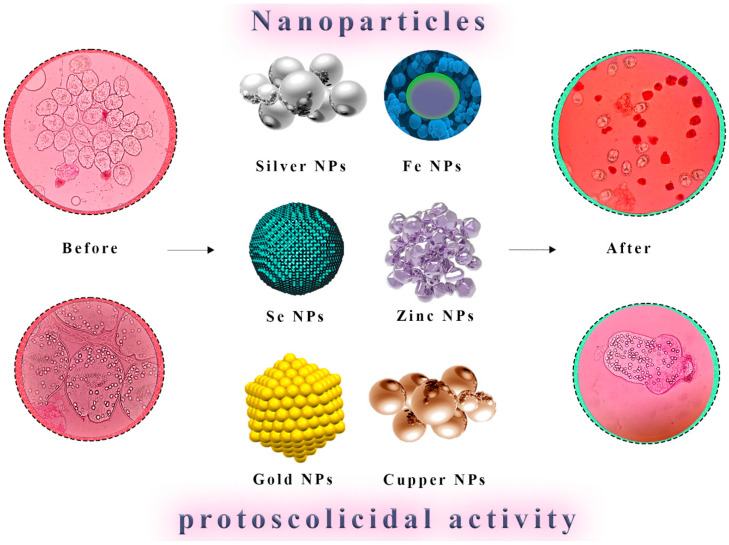
Direct Nanoparticle-Mediated Destruction of Hydatid Cyst Protoscoleces. A visual representation illustrates nanoparticles’ direct application for the targeted destruction of protoscoleces within hydatid cysts. Nanoparticles are shown interacting with protoscoleces, leading to their effective neutralization and elimination. This approach represents a promising avenue for the selective and efficient eradication of hydatid cysts.

**Figure 5:**
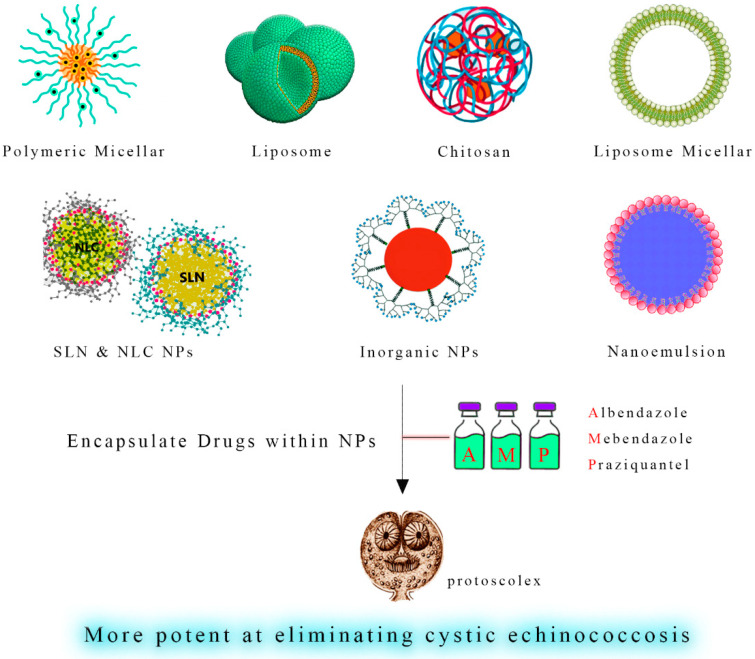
Enhanced Hydatid Cyst Treatment through Nano-Coatings. A schematic depiction showcases nano-coatings as a delivery platform for anthelmintic drugs such as albendazole, mebendazole, and praziquantel. These nano-coatings enable controlled and sustained release of the therapeutic agents, enhancing their action against hydatid cysts while minimizing systemic side effects. This innovative strategy can potentially optimize chemotherapy’s efficacy in managing hydatid cyst infections.

Soltani et al. (2017) have reported that solid lipid nanoparticles loaded with albendazole and albendazole sulfoxide showed superior physicochemical and controlled release properties, indicating that these particles can act as highly efficient drug delivery particles [19]. These findings suggest that such materials might be beneficial for treating cystic echinococcosis. The synergistic administration of praziquantel-loaded nanocarriers with albendazole and mebendazole nanoformulations may improve treatment outcomes and compromise the emergence of drug resistance in hydatid cysts (Figure 4 and Figure 5) (Table 1).

## 6. Nanotechnology Chemotherapy Challenges

This section will present a more comprehensive overview of the concerns that can arise when nanotherapeutics are applied to treat this parasitic disease, including issues related to the potential for toxicity, biodistribution, and the resistance of nanotherapeutics.

### 6.1. Bio-Distribution: Navigating the Intricacies

The biological landscape presents an intricate pathway for nanotherapeutics, so researchers must consider that when deploying them [142,143]. In contrast to commonly used medications, nanoparticles can easily pass through biological barriers because of their small size [144]. The ability to access target sites also necessitates understanding their bio-distribution dynamics, which can be achieved by accessing them [145,146]. A labyrinthine terrain of blood vessels, tissues, and organs must be navigated by nanoparticles that use therapeutic agents to deliver therapeutic agents to hydatid cysts. Obtaining the optimal balance between maximizing accumulation at the cyst site and preventing unintended dissemination to healthy tissues requires the meticulous unraveling of these complexities.

### 6.2. Nanoparticle Toxicity

Despite this, it is imperative to note that nanotherapeutics are attempting to redefine drug delivery, which is paradoxical. Nanoparticles possess the exact qualities that make them effective, but they also carry a risk of toxicity [147,148,149,150]. Material behavior at the nanoscale can differ from its performance at the bulk scale. These divergences might negatively affect biological substances when they interact unexpectedly [151,152]. Questions will inevitably be raised regarding the long-term safety and reliability of nanoparticles administered systemically as a treatment for hydatid cysts. Are these nanoparticles capable of causing inflammatory reactions? Do they tend to damage cells? Before deploying nanoparticles for therapeutic purposes, researchers need to conduct rigorous studies to ensure that there will not be any unwanted effects on the body due to the process.

### 6.3. Nanoparticles Resistance

In the same way that the development of resistance challenges conventional therapies, this problem also impacts nanotherapeutics [122]. Evidence shows that the interactions between biological systems and nanoparticles contribute to developing resistance mechanisms within these systems due to the environment created by these interactions [153,154]. As microbes can evolve to counteract antibiotics with the help of nanoparticles, the interaction between nanoparticles and cellular pathways could alter drug susceptibility [155]. Nanoparticles interact with cellular components in complex ways, underscoring the need for nuanced understanding. For nanotherapeutics to remain effective against hydatid cysts over the long term, efforts are needed to circumvent or manage resistance.

### 6.4. Intricacies of Design: Tailoring for Efficacy

A delicate balance must be struck when designing nanotherapeutics because precision and complexity must be balanced simultaneously [156]. Selecting the suitable drug size, and surface properties for nanoparticles requires considering various factors that can directly affect therapeutic efficacy [157,158,159].

**Figure 6:**
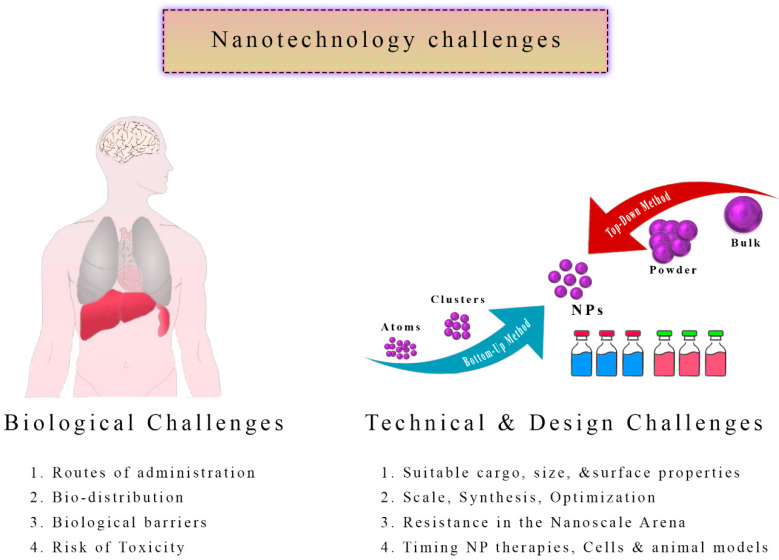
Challenges in the Application of Nanotechnology for Hydatid Cyst Management. An illustrative representation highlights the hurdles and complexities associated with using nanotechnology to treat hydatid cysts. Challenges such as nanoparticle toxicity, scalability, regulatory considerations, and cost-effectiveness are depicted, emphasizing the need for careful evaluation and innovative solutions to harness the full potential of nanotechnology while addressing these limitations. This figure underscores the importance of overcoming these challenges to advance the field of hydatid cyst therapy.

By creating nanoparticles that can cross the physical barriers unique to each patient’s anatomy, a new level of complexity has been added to treating hydatid cysts. For nanotherapeutics to be customized for individual patients, novel strategies must be developed that are both scalable and practical [160,161]. These design intricacies must be overcome if hydatid cysts are to be treated with nanotechnology (Figure 6).

## 7. Nanobiosensors for Early Diagnosis and Treatment Effectiveness

Nanotechnology has catalyzed breakthroughs in medical diagnosis and treatment that were once impossible [162,163]. An advancement within this frontier is nanobiosensors, which promise to revolutionize how diseases are detected and how treatments are optimized [164,165]. A new era of early diagnosis and heightened treatment efficacy for hydatid cysts is ushered in by nanobiosensors, as this section explores them in depth.

Despite their role in hydatid cyst disease, *E. granulosus* larvae have had a deceptive multifocal presentation, which has confounded diagnosis. In current knowledge, nanobiosensors are potent tools that can detect disease-associated biomarkers in complex environments, including bodily fluids, with exceptional specificity and sensitivity [166,167]. To see specific biomolecules intricately linked to hydatid cysts, scientists have developed nanobiosensors that exploit the properties of nanomaterials, such as gold nanoparticles, quantum dots, and carbon nanotubes [54,168,169]. Increasing specificity has led to a paradigm shift in diagnostic accuracy and early detection.

One of the essential reasons that nanobiosensors have such great potency is that they inherit an intrinsic nature amplified by their particular characteristics [170]. The exceptional surface area-to-volume ratio, with excellent electrical conductivity and distinctive optical features, constitutes the sensors’ outstanding performance [171]. By combining these attributes, molecular interactions can be seamlessly converted into discernible signals that can be magnified and meticulously analyzed. A vital outcome of this capability is the ability to monitor cyst progression and therapeutic responses in real-time. Beyond diagnostics, this capability extends to many other applications [172,173].

By extending the boundaries of diagnostics into the realm of personalized medicine, nanobiosensors are enhancing the treatment of cystic hydatid disease. Antiparasitic drug development can be accelerated by monitoring cellular responses in real time, and treatment regimens can be tailored to each patient’s distinctive biological response [174].

This approach will likely improve the efficacy of the treatment while minimizing the possibility of adverse effects. Due to the complex life cycle of hydatid cysts, this approach holds tremendous promise for enhancing effectiveness and minimizing adverse effects. One of the most transformative aspects of this technology is its seamless integration into point-of-care systems, bridging the diagnostic laboratory and immediate patient care gap. The portability and miniaturization of nanobiosensors have permitted their use even in resource-constrained settings where hydatid cyst disease is prevalent. A more accessible diagnostic capability could revolutionize disease management, allowing for more timely interventions, informed decision making, and reduced disease burdens. Despite the persistent challenges of hydatid cyst disease, nanobiosensors are emerging as a promising innovation into diagnosis methods. These nanotechnology-based sensors may reshape hydatid cyst management by providing early detection, individualized treatment approaches, and improved outcomes for infected patients.

## 8. Discussion

The efficacy of traditional chemotherapy for hydatid cysts has been compromised due to several obstacles [175]. A major hurdle is a cyst’s tendency to develop within complicated anatomical sites that are often difficult to reach in the body. Insufficient drug concentrations are, therefore, present on cyst surfaces when chemotherapeutic agents are administered systemically, leading to unsatisfactory treatment outcomes in many cases [176]. By prescribing higher doses of drugs, clinicians risk negatively affecting the health of their patients.

A dense, layered membrane surrounds the hydatid cyst, serving as a barrier of protection [177]. This protective barrier restricts therapeutic agents’ access to the cyst contents, reducing drug exposure. As a result, therapeutic effects are diminished. As an innovative way of overcoming these difficulties, nanotechnology presents an opportunity to improve drug delivery systems [178]. As nanocarriers encapsulate chemotherapeutic agents, they can make them more soluble, stable, and bioavailable, making it possible to administer drugs directly to cysts more precisely and efficiently [179].

Treatment success depends on the early detection and accurate diagnosis of hydatid cysts. Currently, ultrasound and computed tomography are the most commonly used diagnostic techniques, along with serology tests complementing them [180]. These established techniques, nevertheless, have many drawbacks [181]. There is a potential for false negative results or an incomplete characterization of cysts when imaging does not provide a clear and comprehensive view. On the other hand, serological tests do not have the sensitivity or accuracy needed for early detection. [139].

A promising treatment option in hydatid cyst chemotherapy is underway due to nanotechnology’s integration [182]. Nanotechnology aims to improve conventional chemotherapy approaches by addressing their inherent deficiencies [183,184]. This is the driving force for nanotechnology’s development [137,138]. Chemotherapeutic agents can now be encapsulated in nanocarriers, enabling a new era of drug delivery optimization [140,141]. Nanocarriers solve drug solubility issues, and their stability increases therapeutic agents’ bioavailability [185].

One of the most significant aspects of this advancement involves targeted drug delivery to cyst sites. Specifically, labyrinthine vascular pathways can be traversed by nanocarriers efficiently transporting the drugs throughout cysts [186]. Doing so makes it possible to provide superior, more specific, and less harmful remedies [187]. A significant concern of conventional chemotherapy concerns the emergence of resistance, an issue that can be addressed via nanocarriers capable of controlled release [188]. The life of therapeutic drugs within cysts may be prolonged by incorporating nanocarriers to delay the development of resistance processes, opening the door to more durable and effective treatment options.

It is clear that nanotechnology holds promise for the chemotherapy of hydatid cysts, but it is also not without challenges. Nanomaterials can cause health problems primarily due to their toxic properties [189]. The long-term consequences of nanoparticles in humans need thorough safety evaluations and comprehensive biocompatibility trials. From the beginning of the formulation process to the end of the application period of nanotherapeutics, the safety and well-being of patients must remain a priority [190]. Furthermore, hydatid cysts that tend to develop resistance to nanotherapeutics require close monitoring. Different drug classes should be combined in a combination therapy, or nanocarriers loaded with multiple drugs should be explored to prevent this problem. As resistance processes are likely to evolve and treatments must be adapted accordingly, research must be conducted consistently.

As a pioneering tool to enhance hydatid cyst identification and management, nanobiosensors offer exciting possibilities. Biomarkers that indicate diseases can be identified in minute quantities, making them extremely useful for patients, as early diagnosis allows them to begin treatment at the earliest opportunity. Moreover, these nanobiosensors could assist in tailoring treatment based on monitoring therapy effectiveness and detecting relapses early. Thus, it is imperative to realize that designing and optimizing nanobiosensors for a clinical application requires great expertise. Efforts must be made to conduct comprehensive investigation and development, establish protocols, and conduct diligent validation to guarantee that nanobiosensors used in healthcare are trustworthy, accurate, and safe.

## 9. Conclusions

Through the combination of nanotechnology and hydatid cyst chemotherapy and diagnostics, numerous applications exist for improving the treatment of patients with hydatid cysts. As a result of addressing the formidable challenges posed by conventional approaches, nanotechnology can deliver precision drugs, reduce toxicity, and enhance treatment efficacy. In recent years, nanobiosensors have developed into a beacon for early and accurate disease diagnosis, heralding a proactive era in disease management. Detailed safety assessments, meticulous research, and creative strategies to minimize resistance are necessary for optimizing the capabilities of nanotechnology. Hydatid cyst treatment options must be assessed and innovated at a crossroads between scientific discoveries and clinical tests. This journey will improve the management and prognosis for hydatid cyst disease, ultimately improving the quality of life for those affected.

## 10. Future Directions

Using nanotechnology to treat hydatid cysts in the future will offer several possible avenues for research and advancement. With the development of many distinct paths for treating hydatid cysts, the field can build on the progress already made. Future developments will depend on the formulation of nanoparticles according to specific requirements. The surface charge, size, and composition of nanoparticles can be understood when they are tailor-made to maximize their targeting precision and minimize their toxic effects. It may be possible for us to develop new ways to deliver therapeutic agents precisely when and where they are needed when we collaborate on this endeavor.

The development of innovative drug delivery strategies will drive future research. Nanoparticles can respond to specific cues within cyst microenvironments, offering an exciting therapeutic approach. A controlled drug release mechanism could be developed to deliver therapeutic agents precisely to cyst locations to increase cyst treatment effectiveness and minimize collateral damage to healthy tissues. The anti-resistance payloads on nanoparticles may prevent resistance mechanisms from forming in addition to offering chemotherapeutic agents. Through this innovative approach, a multipronged attack is being waged against drug-resistant strains, which is expected to maximize their efficiency.

To develop them, the long-term toxicity and biodistribution of nanotherapeutics must be thoroughly understood. To ensure the safety and effectiveness of nanotechnology-based interventions, rigorous studies must be conducted in these areas. These interventions can improve translational effectiveness by identifying potential side effects and optimizing nanoparticle biodistribution. In addition to nanotherapeutics, conventional drugs or complementary treatments can be incorporated into a nanotherapeutic approach. Nanoparticles can also be combined with established treatment approaches to overcome previously insurmountable obstacles and improve patients’ well-being.

A more widespread use of nanotechnology will likely enable personalized treatment strategies. It is important to consider each patient’s disease profile when designing nanoparticle formulations based on their unique characteristics to improve the treatment’s efficacy. Through precision medicine practices and hydatid cyst management, therapeutic interventions can be aligned with the patient’s needs, bringing a new era in hydatid cyst management.

Collaboration is becoming increasingly important in nanotechnology, which is nearing clinical translation. Translating laboratory discoveries into clinical applications requires researchers, clinicians, and regulatory agencies’ collaboration. In considering interdisciplinary regulatory considerations and ethical concerns related to nanotechnology-based therapies, it is important to consider the complex regulatory and moral issues involved. It is also important to follow up for long periods to determine whether nanotherapeutics are effective and how they impact patients’ health. The long-term monitoring of patients’ trajectories is recommended to determine whether treatment effects are durable over time.

It will be fascinating to watch the trajectory of nanobiosensors on the diagnostic front in the future. Hydatid cyst detection would be revolutionized by these sensors in the early stages of cyst development, possibly saving lives. Multiplexed sensors that detect multiple biomarkers associated with the disease will be developed to improve diagnostic accuracy and speed. This would ultimately result in quicker and more accurate treatment. A significant transformation has occurred in point-of-care diagnostics thanks to incorporating nanobiosensors. In situations with limited resources, these portable devices can provide fast and accurate results, especially when speed and accuracy are essential. Consequently, this new development could fundamentally change the disease detection and intervention landscape.

Combining advanced imaging techniques with nanotechnology is another avenue to explore. Integrating nanoparticles and cutting-edge imaging technologies such as MRI and PET could allow for the real-time monitoring of treatment responses and disease progression. Adapting and fine-tuning the treatment based on the patient’s needs as it evolves is a key component of dynamic treatment. Global collaboration remains the overarching need despite these diverse paths. The synergy between researchers, healthcare providers, policymakers, and industry stakeholders can catalyze this domain. Hydatid cyst treatments can be developed more quickly by collaborating across borders, resolving complex challenges, and introducing innovative approaches.

## Figures and Tables

**Table 1:** This table offers a comprehensive overview of the latest advancements in hydatid cyst treatment, where nanotechnology has been harnessed for therapeutic purposes. This table compiles a compilation of studies exploring nanotechnology, whether in controlled laboratory settings (in vitro), within living organisms (in vivo), or through a combination of both approaches. These investigations encompass a wide spectrum of applications, all leveraging various types of nanoparticles and nanocoatings, each designed with distinct properties and mechanisms of action. This compilation serves as a valuable resource for researchers and healthcare professionals, shedding light on the evolving landscape of hydatid cyst therapy through integrating nanoscale technologies.

References	Efficacy Assessment	Treatment Period	Disease	Dosage	Experimental Design	Compounds
[35]	The weight and volume of cysts in the treated group were statistically significant compared with the control group	45 days	CE: Hydatid cyst of *Echinococcus granulosus*	10 mg/kg/day	In vivo	Albendazole sulfoxide-loaded chitosan-PLGA NPs (ABZ-SO-loaded CS-PGLA NPs)
[137]	The treated (ABZ-LNCs) group did not show any cysts	30 days by an intragastric tube	CE	5 mg/kg/day	In vivo	Albendazole-lipid Nanocapsule (ABZ-LNCs)
[20]	High efficacy in experimentally infected mice	8 weeks by an gastric tube	CE	100 mg/kg/day	In vivo	Albendazole on Ag NPs, ABZ, and Ag NPs
[43]	Only at a concentration of 800 μg/mL (100% PSCs mortality rate after 4 days of exposure)	-	CE	200, 400, and 800 μg/mL	In vitro	ABZ-loaded β-cyclodextrin (ABZ-β-CD)
[44]	Both in vitro and in vivo treatments with ABZ-NLCs are significantly more efficient than treatment with free ABZ	Treatment was performed on Balb/C mice 1 day before intraperitoneal injection of viable protoscoleces	CE	1, 5, and 10 μg/mL	In vitro and in vivo	Albendazole-loaded nanostructured lipid (ABZ-NLCs)
[21]	1 µg/mL ABZ-NCs as a scolicidal agent against hydatid cyst protoscolices in 17 and 23 days	5–30 days	CE	Protoscolices were cultured in 1 mL of RPMI 1640+ 1 µg/mL ABZ- nanocrystal and ABZ were added to culture, incubated at 37 °C in 5% CO_2_	In vitro	ABZ nanocrystals (ABZ-NCs)
[28]	Ag NPs showed the highest effect, followed by SiNPs, CuNPs, FeNPs, and ZnNPs	10–60 min	CE	0.25, 0.5, and 1 mg/mL	In vitro	Ag NPs, Fe NPs, Cu NPs, Si NPs, and Zn NPs
[37]	47.8% after 45 min mortality rate of the protoscolices, in-creased from 10.4% after 15 min to 47.8% after 45 min.	15, 30, and 45 min	CE	10 μg/mL	In vitro	AgNPs
[36]	The cysts in the treated animals were slightly smaller, the weight of infected treated mice was more reduced than those in the non-treated control group	Treatment every 2 days for 30 days, orally administered, BALB/c Mice	CE	50, 100, 200, and 300 mg/kg AgNPs diluted in distilled water	In vivo	Green synthesis of silver nanoparticles using Z. spina-christi (sidr) leaf extract
[138]	The mortality rate was 68% in 4 mg/mL concentration	5, 10, 20, 30, and 60 min	CE	0.25, 0.05, 1, 2, and 4 mg/mL,	In vitro	Chitosan–Curcumin Nanoparticles
[135]	Mortality rate was 100% after 10 min of incubation with 750 mg/mL of CuNPs and with Albendazole	5–60 min	CE	CuNPs 250, 500, and 750 mg/mL and Albendazole 200 mg/mL	In vitro and ex vivo	Copper NPs (CuNPs)
[139]	The effect of concentrations of 250 and 500 mg/mL was the greatest and most clear since the first ten minutes of exposure	10, 30, and 60 min	CE	50, 125, 250, and 500 micrograms/mL	In vitro	Copper(core-shell) Nanoparticles
[38]	Mortality of the protoscoleces was 100% after 120 min of exposure to 1250 and 625 µg/mL concentrations of CUR-NE	10, 20, 30, 60, and 120 min	CE	156, 312, 625, and 1250 µg/mL	In vitro	Curcumin nanoemulsion (CUR-NE)
[117]	4000 μg/mL of gold NPs killed 76% of protoscoleces in 60 min	5, 10, 20, 30, and 60 min	CE	250, 500, 1000, 2000, and 4000 μg/mL	In vitro	Gold NPs
[41]	Mortality rate was 100% after 2 h of incubation with Ag NPs 0.4 mg/L	10–120 min	CE	0.05, 0.1, 0.2, 0.3, and 0.4 mg/mL	In vitro	Silver Nanoparticles Ag NPs
[140]	At 20 min, SA-ZnO-NPs at 2000 μg/mL exhibited the greatest activity on protoscolices with 100% mortality	10, 20, and 30 min	CE	1500 and 2000 μg/mL	In vitro	Salicylate-coated Zinc oxide nanoparticles (SA-ZnO-NPs)
[39]	Killed 84% of the treated protoscolices	60 min	CE	15 μg/mL gonad extract + TiO_2_ Nanoparticles	In vivo and In vitro	TiO_2_ Nanoparticles and Echinometra mathaeis gonad extracts
[42]	The mortality rate of 50 mg/mL ZnO NPs is 19.6% of protoscolices at 10 min	10, 30, and 60 min	CE	50, 100, and 150 mg/mL	In vitro	Zinc oxide Nanoparticles ZnO-NPs
[126]	1000, 2000, and 4000 μg/mL were significantly effective in the killing of protoscoleces	60 min	CE	250, 500, 1000, 2000, and 4000 μg/mL	In vitro	Zirconium Oxide (ZrO_2_)
[141]	The concentration of 200 μg/mL, completely killed the protoscolices after 10 min	50, 100, and 200 μg/mL) alone and combined with albendazole (ALZ, 100 μg/mL)	CE	50, 100, and 200 μg/mL)	In vitro and ex vivo	ZnNPs

## Data Availability

The datasets generated during the current study are available from the corresponding author upon reasonable request.

## References

[1] Lupia T., Corcione S., Guerrera F., Costardi L., Ruffini E., Pinna S.M., Rosa F.G.D. (2021). Pulmonary echinococcosis or lung hydatidosis: A narrative review. Surg. Infect..

[2] Gessese A.T. (2020). Review on epidemiology and public health significance of hydatidosis. Vet. Med. Int..

[3] Karshima S.N., Ahmed M.I., Adamu N.B., Magaji A.A., Zakariah M., Mohammed K. (2022). Africa-wide meta-analysis on the prevalence and distribution of human cystic echinococcosis and canine *Echinococcus granulosus* infections. Parasit. Vectors.

[4] Alvi M.A., Alsayeqh A.F. (2022). Food-borne zoonotic echinococcosis: A review with special focus on epidemiology. Front. Vet. Sci..

[5] Casulli A., Massolo A., Saarma U., Umhang G., Santolamazza F., Santoro A. (2022). Species and genotypes belonging to *Echinococcus granulosussensu lato* complex causing human cystic echinococcosis in Europe (2000–2021): A systematic review. Parasit. Vectors.

[6] Sadr S., Charbgoo A., Borji H., Hajjafari A. (2022). Interactions between innate immunity system and *Echinococcus granulosus*: Permission for vaccine development. Ser. Med. Sci..

[7] Díaz Á. (2017). Immunology of cystic echinococcosis (hydatid disease). Br. Med. Bull..

[8] Hidalgo C., Stoore C., Strull K., Franco C., Corrêa F., Jiménez M., Hernández M., Lorenzatto K., Ferreira H.B., Galanti N. (2019). New insights of the local immune response against both fertile and infertile hydatid cysts. PLoS ONE.

[9] Díaz A., Casaravilla C., Irigoín F., Lin G., Previato J.O., Ferreira F. (2011). Understanding the laminated layer of larval Echinococcus I: Structure. Trends. Parasitol..

[10] Goussard P., Eber E., Mfingwana L., Nel P., Schubert P., Janson J., Pitcher R., le Roux C. (2022). Paediatric pulmonary echinococcosis: A neglected disease. Paediatr. Respir. Rev..

[11] Brehm K., Koziol U. (2017). Echinococcus—Hst interactions at cellular and molecular levels. Adv. Parasitol..

[12] Thompson R. (2017). Biology and systematics of Echinococcus. Adv. Parasitol..

[13] Sadr S., Poorjafari Jafroodi P., Haratizadeh M.J., Ghasemi Z., Borji H., Hajjafari A. (2023). Current status of nano-vaccinology in veterinary medicine science. Vet. Med. Sci..

[14] Pramanik P.K.D., Solanki A., Debnath A., Nayyar A., El-Sappagh S., Kwak K.-S. (2020). Advancing modern healthcare with nanotechnology, nanobiosensors, and internet of nano things: Taxonomies, applications, architecture, and challenges. IEEE Access.

[15] Sahu T., Ratre Y.K., Chauhan S., Bhaskar L., Nair M.P., Verma H.K. (2021). Nanotechnology based drug delivery system: Current strategies and emerging therapeutic potential for medical science. J. Drug Deliv. Sci. Technol..

[16] Sindhwani S., Chan W.C. (2021). Nanotechnology for modern medicine: Next step towards clinical translation. J. Intern. Med..

[17] Prasad R.D., Charmode N., Shrivastav O.P., Prasad S.R., Moghe A., Sarvalkar P.D., Prasad N.R. (2021). A review on concept of nanotechnology in veterinary medicine. ES Food Agrofor..

[18] Shnawa B.H., Al-Ali S.J., Swar S.O. (2021). Nanoparticles as a new approach for treating hydatid cyst disease. Veterinary Pathobiology and Public Health.

[19] Soltani S., Rafiei A., Ramezani Z., Abbaspour M.R., Jelowdar A., Kahvaz M.S. (2017). Evaluation of the hydatid cyst membrane permeability of albendazole and albendazole sulfoxide-loaded solid lipid nanoparticles. Jundishapur J. Nat. Pharm. Prod..

[20] Nassef N.E., Saad A.-G.E., Harba N.M., Beshay E.V., Gouda M.A., Shendi S.S., Mohamed A.S.E.-D. (2019). Evaluation of the therapeutic efficacy of albendazole-loaded silver nanoparticles against *Echinococcus granulosus* infection in experimental mice. J. Parasit. Dis..

[21] Fateh R., Norouzi R., Mirzaei E., Nissapatron V., Nawaz M., Khalifeh-Gholi M., Hamta A., Sadati S.J.A., Siyadatpanah A., Bafghi A.F. (2021). In vitro evaluation of albendazole nanocrystals against *Echinococcus granulosus* protoscolices. Ann. Parasitol..

[22] Maurice M.N., Huseein E.A.M., Monib M.E.S.M., Alsharif F.M., Namazi N.I., Ahmad A.A. (2021). Evaluation of the scolicidal activities of eugenol essential oil and its nanoemulsion against protoscoleces of hydatid cysts. PLoS ONE.

[23] Alsharedeh R.H., Rezigue M., Bashatwah R.M., Amawi H., Aljabali A.A., Obeid M.A., Tambuwala M.M. (2023). Nanomaterials as a Potential Target for Infectious Parasitic Agents. Curr. Drug Deliv..

[24] Shnawa B.H., Jalil P.J., Aspoukeh P., Mohammed D.A., Biro D.M. (2022). Protoscolicidal and Biocompatibility Properties of Biologically Fabricated Zinc Oxide Nanoparticles Using *Ziziphus spina*-*christi* Leaves. Pak. Vet. J..

[25] Rafiei A., Soltani S., Ramezani Z., Abbaspour M.R., Jelowdar A., Kahvaz M.S. (2019). Ultrastructural changes on fertile and infertile hydatid cysts induced by conventional and solid lipid nanoparticles of albendazole and albendazole sulfoxide. Comp. Clin. Path..

[26] Çolak B., Aksoy F., Yavuz S., Demircili M.E. (2019). Investigating the effect of gold nanoparticles on hydatid cyst protoscolices under low-power green laser irradiation. Turk. J. Surg..

[27] Aminpour S., Rafiei A., Jelowdar A., Kouchak M. (2019). Evaluation of the protoscolicidal effects of albendazole and albendazole loaded solid lipid nanoparticles. Iran. J. Parasitol..

[28] Norouzi R., Ataei A., Hejazy M., Noreddin A., El Zowalaty M.E. (2020). Scolicidal effects of nanoparticles against hydatid cyst protoscolices in vitro. Int. J. Nanomed..

[29] Ahmadpour E., Godrati-Azar Z., Spotin A., Norouzi R., Hamishehkar H., Nami S., Heydarian P., Rajabi S., Mohammadi M., Perez-Cordon G. (2019). Nanostructured lipid carriers of ivermectin as a novel drug delivery system in hydatidosis. Parasit. Vectors.

[30] Taha R.H. (2022). Green synthesis of silver and gold nanoparticles and their potential applications as therapeutics in cancer therapy. A review. Inorg. Chem. Commun..

[31] Kohansal K., Rafiei A., Kalantari H., Jelowdar A., Salimi A., Rezaie A., Jalali M.R. (2022). Nephrotoxicity of Albendazole and Albendazole Loaded Solid Lipid Nanoparticles in Mice with Experimental Hydatidosis. Adv. Pharm. Bull..

[32] Shnawa B.H., Hamad S.M., Barzinjy A.A., Kareem P.A., Ahmed M.H. (2021). Scolicidal activity of biosynthesized zinc oxide nanoparticles by *Mentha longifolia* L. leaves against *Echinococcus granulosus* protoscolices. Emergent. Mater..

[33] Kishik S., Nagati I., El Hayawan I., Ali I., Fawzy M., Ali H. (2020). Efficacy of Nigella sativa oil and its chitosan loaded nanoparticles on experimental cystic echinoncoccosis with immunological assessment. Parasitol. United J..

[34] Albalawi A.E., Alanazi A.D., Baharvand P., Sepahvand M., Mahmoudvand H. (2020). High potency of organic and inorganic nanoparticles to treat cystic echinococcosis: An evidence-based review. Nanomaterials.

[35] Darvishi M.M., Moazeni M., Alizadeh M., Abedi M., Tamaddon A.M. (2020). Evaluation of the efficacy of albendazole sulfoxide (ABZ-SO)–loaded chitosan-PLGA nanoparticles in the treatment of cystic echinococcosis in laboratory mice. Parasitol. Res..

[36] Hamad S.M., Shnawa B.H., Jalil P.J., Ahmed M.H. (2022). Assessment of the therapeutic efficacy of silver nanoparticles against secondary cystic echinococcosis in BALB/c mice. Surfaces.

[37] Salih T.A., Hassan K.T., Majeed S.R., Ibraheem I.J., Hassan O.M., Obaid A. (2020). In vitro scolicidal activity of synthesised silver nanoparticles from aqueous plant extract against *Echinococcus granulosus*. Biotechnol. Rep..

[38] Teimouri A., Jafarpour Azami S., Hashemi Hafshejani S., Ghanimatdan M., Bahreini M.S., Alimi R., Sadjjadi S.M. (2023). Protoscolicidal effects of curcumin nanoemulsion against protoscoleces of *Echinococcus granulosus*. BMC Complement. Med. Ther..

[39] Navvabi A., Homaei A., Khademvatan S., Ansari M.H.K., Keshavarz M. (2019). Combination of TiO_2_ nanoparticles and *Echinometra mathaeis* gonad extracts: In vitro and in vivo scolicidal activity against hydatid cysts. Biocatal. Agric. Biotechnol..

[40] Firouzeh N., Eslaminejad T., Shafiei R., Faridi A., Fasihi Harandi M. (2021). Lethal in vitro effects of optimized chitosan nanoparticles against protoscoleces of *Echinococcus granulosus*. J. Bioact. Compat. Polym..

[41] Jalil P.J., Shnawa B.H., Hamad S.M. (2021). Silver Nanoparticles: Green Synthesis, Characterization, Blood Compatibility and Protoscolicidal Efficacy against *Echinococcus granulosus*. Pak. Vet. J..

[42] Norouzi R., Hejazy M., Ataei A. (2019). Scolicidal activity of zinc oxide nanoparticles against hydatid cyst protoscolices in vitro. Nanomed. Res. J..

[43] Bakhtiar N.M., Akbarzadeh A., Ahmadpour E., Mahami-Oskouei M., Casulli A., Norouzi R., Asadi M., Ebrahimi M., Asadi N., Oliveira S.M.R. (2022). In vitro efficacy of albendazole-loaded β-cyclodextrin against protoscoleces of *Echinococcus granulosus* sensu stricto. Exp. Parasitol..

[44] Farhadi M., Haniloo A., Rostamizadeh K., Ahmadi N. (2021). In vitro evaluation of albendazole-loaded nanostructured lipid carriers on *Echinococcus granulosus* microcysts and their prophylactic efficacy on experimental secondary hydatidosis. Parasitol. Res..

[45] Kishik S., Nagati I., Ali I., Aly N., Fawzy M., Ali H. (2021). Pathological assessment of Nigella sativa oil and its chitosan loaded nanoparticles on experimental hepatic cystic echinoncoccosis. Parasitol. United J..

[46] Hamid L., Alsayari A., Tak H., Mir S.A., Almoyad M.A.A., Wahab S., Bader G.N. (2023). An Insight into the Global Problem of Gastrointestinal Helminth Infections amongst Livestock: Does Nanotechnology Provide an Alternative?. Agriculture.

[47] Liang W., Wang X.-C., Wu X.-W., Zhang S.-J., Sun H., Ma X., Peng X.-Y. (2014). Efficacy of albendazole chitosan microspheres against *Echinococcus granulosus* infection in mice. Zhongguo Ji Sheng Chong Xue Yu Ji Sheng Chong Bing Za Zhi.

[48] Saeedan M.B., Aljohani I.M., Alghofaily K.A., Loutfi S., Ghosh S. (2020). Thoracic hydatid disease: A radiologic review of unusual cases. World. J. Clin. Cases.

[49] Barnes T.S., Deplazes P., Gottstein B., Jenkins D., Mathis A., Siles-Lucas M., Torgerson P.R., Ziadinov I., Heath D.D. (2012). Challenges for diagnosis and control of cystic hydatid disease. Acta Trop..

[50] Rasheed K., Zargar S.A., Telwani A.A. (2013). Hydatid cyst of spleen: A diagnostic challenge. N. Am. J. Med. Sci..

[51] Garg M.K., Sharma M., Gulati A., Gorsi U., Aggarwal A.N., Agarwal R., Khandelwal N. (2016). Imaging in pulmonary hydatid cysts. World J. Radiol..

[52] Inayat F., Azam S., Baig A.S., Nawaz G., Ullah W. (2019). Presentation patterns, diagnostic modalities, management strategies, and clinical outcomes in patients with hydatid disease of the pelvic bone: A comparative review of 31 cases. Cureus.

[53] Jafari F., Maghsood A.H., Fallah M., Jalilvand A., Matini M., Amini B. (2022). Design of highly sensitive nano-biosensor for diagnosis of hydatid cyst based on gold nanoparticles. Photodiagnosis Photodyn. Ther..

[54] Jain U., Shakya S., Saxena K. (2021). Nano-Biosensing Devices Detecting Biomarkers of Communicable and Non-communicable Diseases of Animals. Biosensors in Agriculture: Recent Trends and Future Perspectives.

[55] Cerbu C., White J.C., Sabliov C.M. (2023). Nanotechnology in livestock: Improving animal production and health. Nano-Enabled Sustainable and Precision Agriculture.

[56] Hassan A.A., Sayed-ElAhl R.M., El Hamaky A.M., Mansour M.K., Oraby N.H., Barakat M.H. (2022). Nanodiagnostics: New Tools for Detection of Animal Pathogens. Nanorobotics and Nanodiagnostics in Integrative Biology and Biomedicine.

[57] Chai J.-Y., Jung B.-K., Hong S.-J. (2021). Albendazole and mebendazole as anti-parasitic and anti-cancer agents: An update. Korean J. Parasitol..

[58] Ün M., Yaman S.S., Erbaş O. (2020). Hydatid cyst and treatment. Demiroglu Sci. Univ. Florence Nightingale J. Transplant..

[59] Adetunji C.O., Egbuna C., Oladosun T.O., Akram M., Micheal O.S., Olisaka F.N., Ozolua P., Adetunji J.B., Enoyoze G.E., Olaniyan O.T. (2021). Efficacy of Phytochemicals of Medicinal Plants for the Treatment of Human Echinococcosis: Echinococcal Disease, Hydatidosis, or Hydatid Disease Drug Discovery. Neglected Tropical Diseases and Phytochemicals in Drug Discovery.

[60] Erfani A., Shahriarirad R., Eskandarisani M., Rastegarian M., Sarkari B. (2023). Management of Liver Hydatid Cysts: A Retrospective Analysis of 293 Surgical Cases from Southern Iran. J. Trop. Med..

[61] Alvi M.A., Khan S., Ali R.M.A., Qamar W., Saqib M., Faridi N.Y., Li L., Fu B.-Q., Yan H.-B., Jia W.-Z. (2022). Herbal medicines against hydatid disease: A systematic review (2000–2021). Life.

[62] Muqaddas H., Mehmood N., Ahmed F., Fatima M., Rasool M., Zafar S., Riaz A., Nauman M. (2023). Problems and perspectives related to cystic echinococcosis in Pakistan: Solutions in one health context. One Health Triad Unique Sci. Publ. Faisalabad Pak..

[63] Wen H., Vuitton L., Tuxun T., Li J., Vuitton D.A., Zhang W., McManus D.P. (2019). Echinococcosis: Advances in the 21st century. Clin. Microbiol. Rev..

[64] Kern P., Da Silva A.M., Akhan O., Müllhaupt B., Vizcaychipi K., Budke C., Vuitton D. (2017). The echinococcoses: Diagnosis, clinical management and burden of disease. Adv. Parasitol..

[65] Taghipour A., Ghaffarifar F., Horton J., Dalimi A., Sharifi Z. (2021). *Silybum marianum* ethanolic extract: In vitro effects on protoscolices of *Echinococcus granulosus* G1 strain with emphasis on other Iranian medicinal plants. Trop. Med. Health.

[66] Jacob S.S., Cherian S., Sumithra T., Raina O., Sankar M. (2013). Edible vaccines against veterinary parasitic diseases—Current status and future prospects. Vaccine.

[67] Wang D., Wu J., Wang Y., Ji Y. (2020). Finding high-quality groundwater resources to reduce the hydatidosis incidence in the Shiqu County of Sichuan Province, China: Analysis, assessment, and management. Expos. Health.

[68] Nunnari G., Pinzone M.R., Gruttadauria S., Celesia B.M., Madeddu G., Malaguarnera G., Pavone P., Cappellani A., Cacopardo B. (2012). Hepatic echinococcosis: Clinical and therapeutic aspects. World J. Gastroenterol..

[69] Kuzucu A., Ulutas H., Reha Celik M., Yekeler E. (2014). Hydatid cysts of the lung: Lesion size in relation to clinical presentation and therapeutic approach. Surg. Today.

[70] Thys S., Sahibi H., Gabriël S., Rahali T., Lefèvre P., Rhalem A., Marcotty T., Boelaert M., Dorny P. (2019). Community perception and knowledge of cystic echinococcosis in the High Atlas Mountains, Morocco. BMC Public Health.

[71] Lightowlers M.W., Gasser R.B., Hemphill A., Romig T., Tamarozzi F., Deplazes P., Torgerson P.R., Garcia H.H., Kern P. (2021). Advances in the treatment, diagnosis, control and scientific understanding of taeniid cestode parasite infections over the past 50 years. Int. J. Parasitol..

[72] Habibi B., Gholami S., Bagheri A., Fakhar M., Moradi A., Khazeei Tabari M.A. (2023). Cystic echinococcosis microRnas as potential non-invasive biomarkers: Current insights and upcoming perspective. Expert Rev. Mol. Diagn..

[73] Dietrich C.F., Douira-Khomsi W., Gharbi H., Sharma M., Cui X.W., Sparchez Z., Richter J., Kabaalioğlu A., Atkinson N.S., Schreiber-Dietrich D. (2020). Cystic and alveolar echinococcosis of the hepatobiliary tract—The role of new imaging techniques for improved diagnosis. Med. Ultrason..

[74] Noori I.F., Jabbar A.S. (2020). Hepatic Hydatid Cyst Diseases during Pregnancy: Diagnosis, Management and Best Practice. Syst. Rev. Pharm..

[75] Caraiani C., Yi D., Petresc B., Dietrich C. (2020). Indications for abdominal imaging: When and what to choose?. J. Ultrason..

[76] Abbasi B., Akhavan R., Khameneh A.G., Amirkhiz G.D.H., Rezaei-Dalouei H., Tayebi S., Hashemi J., Aminizadeh B., Amirkhiz S.D.H. (2021). Computed tomography and magnetic resonance imaging of hydatid disease: A pictorial review of uncommon imaging presentations. Heliyon.

[77] Calame P., Weck M., Busse-Cote A., Brumpt E., Richou C., Turco C., Doussot A., Bresson-Hadni S., Delabrousse E. (2022). Role of the radiologist in the diagnosis and management of the two forms of hepatic echinococcosis. Insights Imaging.

[78] Tamarozzi F., Silva R., Fittipaldo V.A., Buonfrate D., Gottstein B., Siles-Lucas M. (2021). Serology for the diagnosis of human hepatic cystic echinococcosis and its relation with cyst staging: A systematic review of the literature with meta-analysis. PLoS Negl. Trop. Dis..

[79] Alvi M.A., Ali R.M.A., Khan S., Saqib M., Qamar W., Li L., Fu B.-Q., Yan H.-B., Jia W.-Z. (2023). Past and Present of Diagnosis of Echinococcosis: A Review (1999–2021). Acta Trop..

[80] Meftahi G.H., Bahari Z., Zarei Mahmoudabadi A., Iman M., Jangravi Z. (2021). Applications of western blot technique: From bench to bedside. Biochem. Mol. Biol. Educ..

[81] Alshoabi S.A., Alkalady A.H., Almas K.M., Magram A.O., Algaberi A.K., Alareqi A.A., Hamid A.M., Alhazmi F.H., Qurashi A.A., Abdulaal O.M. (2023). Hydatid disease: A radiological pictorial review of a great neoplasms mimicker. Diagnostics.

[82] Chouhan M., Wiley E., Chiodini P., Amin Z. (2019). Hepatic alveolar hydatid disease (*Echinococcus multilocularis*), a mimic of liver malignancy: A review for the radiologist in non-endemic areas. Clin. Radiol..

[83] Khalili N., Iranpour P., Khalili N., Haseli S. (2023). Hydatid Disease: A Pictorial Review of Uncommon Locations. Iran. J. Med. Sci..

[84] Pakala T., Molina M., Wu G.Y. (2016). Hepatic echinococcal cysts: A review. J. Clin. Transl. Hepatol..

[85] Paramita A.A.K.Y., Wibawa I.D.N. (2023). Multimodal Treatment of Cystic Echinococcosis. Indones. J. Gastroenterol. Hepatol. Dig. Endosc..

[86] Borhani M., Fathi S., Darabi E., Jalousian F., Simsek S., Ahmed H., Kesik H.K., Hosseini S.H., Romig T., Harandi M.F. (2021). Echinococcoses in Iran, Turkey, and Pakistan: Old diseases in the new millennium. Clin. Microbiol. Rev..

[87] Fries C.N., Curvino E.J., Chen J.-L., Permar S.R., Fouda G.G., Collier J.H. (2021). Advances in nanomaterial vaccine strategies to address infectious diseases impacting global health. Nat. Nanotechnol..

[88] Khan S.T., Adil S.F., Shaik M.R., Alkhathlan H.Z., Khan M., Khan M. (2021). Engineered nanomaterials in soil: Their impact on soil microbiome and plant health. Plants.

[89] Arshad R., Gulshad L., Haq I.U., Farooq M.A., Al-Farga A., Siddique R., Manzoor M.F., Karrar E. (2020). Advances and challenges in nanocarriers and nanomedicines for veterinary application. Int. J. Pharm..

[90] Sanabria R. (2021). Nanotechnological improvement of veterinary anthelmintics. Pharm. Nanotechnol..

[91] Fatehbasharzad P., Fatehbasharzad P., Sillanpää M., Shamsi Z. (2021). Investigation of bioimpacts of metallic and metallic oxide nanostructured materials: Size, shape, chemical composition, and surface functionality: A review. Part. Part. Syst. Charact..

[92] Saravanan A., Kumar P.S., Karishma S., Vo D.-V.N., Jeevanantham S., Yaashikaa P., George C.S. (2021). A review on biosynthesis of metal nanoparticles and its environmental applications. Chemosphere.

[93] Hikal W.M., Bratovcic A., Baeshen R.S., Tkachenko K.G., Said-Al Ahl H.A. (2021). Nanobiotechnology for the detection and control of waterborne parasites. Open J. Ecol..

[94] Badirzadeh A., Alipour M., Najm M., Vosoogh A., Vosoogh M., Samadian H., Hashemi A.S., Farsangi Z.J., Amini S.M. (2022). Potential therapeutic effects of curcumin coated silver nanoparticle in the treatment of cutaneous leishmaniasis due to Leishmania major in-vitro and in a murine model. J. Drug Deliv. Sci. Technol..

[95] Zhang P., Gong J., Jiang Y., Long Y., Lei W., Gao X., Guo D. (2023). Application of Silver Nanoparticles in Parasite Treatment. Pharmaceutics.

[96] Ali M., Afzal M., Verma M., Bhattacharya S.M., Ahmad F., Samim M., Abidin M.Z., Dinda A.K. (2014). Therapeutic efficacy of poly (lactic-co-glycolic acid) nanoparticles encapsulated ivermectin (nano-ivermectin) against brugian filariasis in experimental rodent model. Parasitol. Res..

[97] Wang Q., Sun X., Huang X., Huang J., Hasan M.W., Yan R., Xu L., Song X., Li X. (2021). Nanoparticles of Chitosan/Poly (D, L-Lactide-Co-Glycolide) enhanced the immune responses of haemonchus contortus HCA59 antigen in model mice. Int. J. Nanomed..

[98] Li J., Yang Y., Han X., Li J., Tian M., Qi W., An H., Wu C., Zhang Y., Han S. (2023). Oral Delivery of Anti-Parasitic Agent-Loaded PLGA Nanoparticles: Enhanced Liver Targeting and Improved Therapeutic Effect on Hepatic Alveolar Echinococcosis. Int. J. Nanomed..

[99] Spirescu V.A., Chircov C., Grumezescu A.M., Andronescu E. (2021). Polymeric nanoparticles for antimicrobial therapies: An up-to-date overview. Polymers.

[100] Cheraghipour K., Rouzbahani A.K., Fallahi S., Taherpour F., Moradifard F., Shakib P., Lashgarian H.E., Marzban A. (2023). Recent Advances in Therapeutic Strategies against Hydatid Cysts using Nanomaterials: A Systematic Review. Lett. Drug Des. Discov..

[101] Mishra D.K., Shandilya R., Mishra P.K. (2018). Lipid based nanocarriers: A translational perspective. Nanomed. NBM..

[102] Sasidharan S., Saudagar P. (2020). Encapsulation and delivery of antiparasitic drugs: A review. Encapsul. Act. Mol. Deliv. Syst..

[103] Yetisgin A.A., Cetinel S., Zuvin M., Kosar A., Kutlu O. (2020). Therapeutic nanoparticles and their targeted delivery applications. Molecules.

[104] Salah E., Abouelfetouh M.M., Pan Y., Chen D., Xie S. (2020). Solid lipid nanoparticles for enhanced oral absorption: A review. Colloids Surf. B.

[105] Cruz A.A., Molento M.B. (2015). Nanotechnology: Meeting the future of Veterinary Parasitology Research. Pesqui. Vet. Bras..

[106] Qadeer A., Ullah H., Sohail M., Safi S.Z., Rahim A., Saleh T.A., Arbab S., Slama P., Horky P. (2022). Potential application of nanotechnology in the treatment, diagnosis, and prevention of schistosomiasis. Front. Bioeng. Biotechnol..

[107] Haby M.M., Sosa Leon L.A., Luciañez A., Nicholls R.S., Reveiz L., Donadeu M. (2020). Systematic review of the effectiveness of selected drugs for preventive chemotherapy for *Taenia solium* taeniasis. PLoS Negl. Trop. Dis..

[108] Hassan N.M., Ghazy A.A. (2022). Advances in diagnosis and control of anthelmintic resistant gastrointestinal helminths infecting ruminants. J. Parasit. Dis..

[109] Arshad R., Gulshad L., Haq I.U., Farooq M.A., Al-Farga A., Siddique R., Manzoor M.F., Karrar E. (2021). Nanotechnology: A novel tool to enhance the bioavailability of micronutrients. Food Sci. Nutr..

[110] Malik S., Muhammad K., Waheed Y. (2023). Nanotechnology: A revolution in modern industry. Molecules.

[111] Wang S., Ma Y., Wang W., Dai Y., Sun H., Li J., Wang S., Li F. (2022). Status and prospect of novel treatment options toward alveolar and cystic echinococcosis. Acta Trop..

[112] Souri M., Soltani M., Kashkooli F.M., Shahvandi M.K. (2022). Engineered strategies to enhance tumor penetration of drug-loaded nanoparticles. J. Control. Release.

[113] Huang M., Zhang M., Zhu H., Du X., Wang J. (2022). Mucosal vaccine delivery: A focus on the breakthrough of specific barriers. Acta Pharm. Sin. B..

[114] Bajwa H.U.R., Khan M.K., Abbas Z., Riaz R., Rehman T., Abbas R.Z., Almutairi M.M., Alshammari F.A., Alraey Y. (2022). Nanoparticles: Synthesis and their role as potential drug candidates for the treatment of parasitic diseases. Life.

[115] Król G., Fortunka K., Majchrzak M., Piktel E., Paprocka P., Mańkowska A., Lesiak A., Karasiński M., Strzelecka A., Durnaś B. (2023). Metallic Nanoparticles and Core-Shell Nanosystems in the Treatment, Diagnosis, and Prevention of Parasitic Diseases. Pathogens.

[116] Boudier A., Le Faou A. (2023). Nanoparticles and Other Nanostructures and the Control of Pathogens: From Bench to Vaccines. Int. J. Mol. Sci..

[117] Napooni S., Arbabi M., Delavari M., Hooshyar H., Rasti S. (2019). Lethal effects of gold nanoparticles on protoscolices of hydatid cyst: In vitro study. Comp. Clin. Pathol..

[118] Jelowdar A., Rafiei A., Abbaspour M.R., Rashidi I., Rahdar M. (2017). Efficacy of combined albendazol and praziquntel and their loaded solid lipid nanoparticles components in chemoprophylaxis of experimental hydatidosis. Asian Pac. J. Trop. Biomed..

[119] Rahimi M.T., Ahmadpour E., Esboei B.R., Spotin A., Koshki M.H.K., Alizadeh A., Honary S., Barabadi H., Mohammadi M.A. (2015). Scolicidal activity of biosynthesized silver nanoparticles against *Echinococcus granulosus* protoscolices. Int. J. Surg..

[120] Lashkarizadeh M.R., Asgaripour K., Dezaki E.S., Harandi M.F. (2015). Comparison of scolicidal effects of amphotricin B, silver nanoparticles, and *Foeniculum vulgare* Mill on hydatid cysts protoscoleces. Iran. J. Parasitol..

[121] Laffleur F., Keckeis V. (2020). Advances in drug delivery systems: Work in progress still needed?. Int. J. Pharm..

[122] Patra J.K., Das G., Fraceto L.F., Campos E.V.R., Rodriguez-Torres M.d.P., Acosta-Torres L.S., Diaz-Torres L.A., Grillo R., Swamy M.K., Sharma S. (2018). Nano-based drug delivery systems: Recent developments and future prospects. J. Nanobiotechnol..

[123] Abidi H., Ghaedi M., Rafiei A., Jelowdar A., Salimi A., Asfaram A., Ostovan A. (2018). Magnetic solid lipid nanoparticles co-loaded with albendazole as an anti-parasitic drug: Sonochemical preparation, characterization, and in vitro drug release. J. Mol. Liq..

[124] Bhatia M., Kumar S., Kapoor A., Lohan S. (2022). A Review on the Drug Delivery Strategies for Parasitic Infections: Scope and Assertion. Drug Deliv..

[125] Sinha S., Sehgal R. (2022). Nano-targeted drug delivery for parasitic infections. Emerging Nanomaterials and Nano-Based Drug Delivery Approaches to Combat Antimicrobial Resistance.

[126] Ibrahim A.A.-J. (2020). Scolicidal activity of zirconium oxide (ZrO_2_) nanoparticles against protoscolices of hydatid cysts. Indian J. Forensic Med. Toxicol..

[127] Joshi G., Quadir S.S., Yadav K.S. (2021). Road map to the treatment of neglected tropical diseases: Nanocarriers interventions. J. Control. Release.

[128] Khalid M., El-Sawy H.S. (2017). Polymeric nanoparticles: Promising platform for drug delivery. Int. J. Pharm..

[129] Kesharwani P., Jain K., Jain N.K. (2014). Dendrimer as nanocarrier for drug delivery. Prog. Polym. Sci..

[130] Mishra V., Bansal K.K., Verma A., Yadav N., Thakur S., Sudhakar K., Rosenholm J.M. (2018). Solid lipid nanoparticles: Emerging colloidal nano drug delivery systems. Pharmaceutics.

[131] Pandian S.R.K., Panneerselvam T., Pavadai P., Govindaraj S., Ravishankar V., Palanisamy P., Sampath M., Sankaranarayanan M., Kunjiappan S. (2021). Nano based approach for the treatment of neglected tropical diseases. Front. Nanotechnol..

[132] Shukla R., Mourya A., Handa M., Ujjwal R.R. (2021). Role of nanomedicines in neglected tropical diseases. Nanopharmaceutical Advanced Delivery Systems.

[133] Khezerlou A., Alizadeh-Sani M., Azizi-Lalabadi M., Ehsani A. (2018). Nanoparticles and their antimicrobial properties against pathogens including bacteria, fungi, parasites and viruses. Microb. Pathog..

[134] Khashan K.S., Sulaiman G.M., Hussain S.A., Marzoog T.R., Jabir M.S. (2020). Synthesis, characterization and evaluation of anti-bacterial, anti-parasitic and anti-cancer activities of aluminum-doped zinc oxide nanoparticles. J. Inorg. Organomet. Polym. Mater..

[135] Ezzatkhah F., Khalaf A.K., Mahmoudvand H. (2021). Copper nanoparticles: Biosynthesis, characterization, and protoscolicidal effects alone and combined with albendazole against hydatid cyst protoscoleces. Biomed. Pharmacother..

[136] Naseri M., Akbarzadeh A., Spotin A., Akbari N.A.R., Mahami-Oskouei M., Ahmadpour E. (2016). Scolicidal and apoptotic activities of albendazole sulfoxide and albendazole sulfoxide-loaded PLGA-PEG as a novel nanopolymeric particle against *Echinococcus granulosus* protoscoleces. Parasitol. Res..

[137] Gamboa G.V., Pensel P.E., Elissondo M.C., Bruni S.F., Benoit J.P., Palma S.D., Allemandi D.A. (2019). Albendazole-lipid nanocapsules: Optimization, characterization and chemoprophylactic efficacy in mice infected with *Echinococcus granulosus*. Exp. Parasitol..

[138] Napooni S., Delavari M., Arbabi M., Barkheh H., Rasti S., Hooshyar H., Mostafa Hosseinpour Mashkani S. (2019). Scolicidal effects of chitosan-curcumin nanoparticles on the hydatid cyst protoscolices. Acta Parasitol..

[139] Deng M.H., Zhong L.Y., Kamolnetr O., Limpanont Y., Lv Z.Y. (2019). Detection of helminths by loop-mediated isothermal amplification assay: A review of updated technology and future outlook. Infect. Dis. Poverty.

[140] Bosetti R., Vereeck L. (2011). Future of nanomedicine: Obstacles and remedies. Nanomedicine.

[141] Devadasu V.R., Bhardwaj V., Kumar M.R. (2013). Can controversial nanotechnology promise drug delivery?. Chem. Rev..

[142] Tirumala M.G., Anchi P., Raja S., Rachamalla M., Godugu C. (2021). Novel methods and approaches for safety evaluation of nanoparticle formulations: A focus towards in vitro models and adverse outcome pathways. Front. Pharmacol..

[143] Roco M.C., Mirkin C.A., Hersam M.C. (2011). Nanotechnology research directions for societal needs in 2020: Summary of international study. J. Nanopart. Res..

[144] Shah S., Famta P., Bagasariya D., Charankumar K., Amulya E., Khatri D.K., Raghuvanshi R.S., Singh S.B., Srivastava S. (2022). Nanotechnology based drug delivery systems: Does shape really matter?. Int. J. Pharm..

[145] Khan I., Saeed K., Khan I. (2019). Nanoparticles: Properties, applications and toxicities. Arab. J. Chem..

[146] Mitchell M.J., Billingsley M.M., Haley R.M., Wechsler M.E., Peppas N.A., Langer R. (2021). Engineering precision nanoparticles for drug delivery. Nat. Rev. Drug Discov..

[147] Hofmann-Amtenbrink M., Grainger D.W., Hofmann H. (2015). Nanoparticles in medicine: Current challenges facing inorganic nanoparticle toxicity assessments and standardizations. Nanomed. Nanotechnol. Biol. Med..

[148] Rehman A.U., Nazir S., Irshad R., Tahir K., ur Rehman K., Islam R.U., Wahab Z. (2021). Toxicity of heavy metals in plants and animals and their uptake by magnetic iron oxide nanoparticles. J. Mol. Liq..

[149] Yin B., Sun W., Zhang X., Liew K. (2022). Deciphering structural biological materials: Viewing from the mechanics perspective and their prospects. Compos. B Eng..

[150] Vega-Vásquez P., Mosier N.S., Irudayaraj J. (2020). Nanoscale drug delivery systems: From medicine to agriculture. Front. Bioeng. Biotechnol..

[151] Bundschuh M., Filser J., Lüderwald S., McKee M.S., Metreveli G., Schaumann G.E., Schulz R., Wagner S. (2018). Nanoparticles in the environment: Where do we come from, where do we go to?. Environ. Sci. Eur..

[152] Prajitha N., Athira S., Mohanan P. (2019). Bio-interactions and risks of engineered nanoparticles. Environ. Res..

[153] Wang L., Hu C., Shao L. (2017). The antimicrobial activity of nanoparticles: Present situation and prospects for the future. Int. J. Nanomed..

[154] Lyshevski S.E. (2018). MEMS and NEMS: Systems, Devices, and Structures.

[155] Chenthamara D., Subramaniam S., Ramakrishnan S.G., Krishnaswamy S., Essa M.M., Lin F.H., Qoronfleh M.W. (2019). Therapeutic efficacy of nanoparticles and routes of administration. Biomater. Res..

[156] Sharma A., Madhunapantula S.V., Robertson G.P. (2012). Toxicological considerations when creating nanoparticle-based drugs and drug delivery systems. Exp. Opin. Drug. Metab. Toxicol..

[157] Panariti A., Miserocchi G., Rivolta I. (2012). The effect of nanoparticle uptake on cellular behavior: Disrupting or enabling functions?. Nanotechnol. Sci. Appl..

[158] Fornaguera C., García-Celma M.J. (2017). Personalized nanomedicine: A revolution at the nanoscale. J. Pers. Med..

[159] Han H.J., Ekweremadu C., Patel N. (2019). Advanced drug delivery system with nanomaterials for personalised medicine to treat breast cancer. J. Drug. Deliv. Sci. Technol..

[160] Kirsch J., Siltanen C., Zhou Q., Revzin A., Simonian A. (2013). Biosensor technology: Recent advances in threat agent detection and medicine. Chem. Soc. Rev..

[161] Luis A.I.S., Campos E.V.R., de Oliveira J.L., Fraceto L.F. (2019). Trends in aquaculture sciences: From now to use of nanotechnology for disease control. Rev. Aquac..

[162] Javaid M., Haleem A., Singh R.P., Rab S., Suman R. (2021). Exploring the potential of nanosensors: A brief overview. Sens. Int..

[163] Jain S., Santana W., Dolabella S.S., Santos A.L., Souto E.B., Severino P. (2021). Are Nanobiosensors an improved solution for diagnosis of leishmania?. Pharmaceutics.

[164] Kim J.H., Suh Y.J., Park D., Yim H., Kim H., Kim H.J., Yoon D.S., Hwang K.S. (2021). Technological advances in electrochemical biosensors for the detection of disease biomarkers. Biomed. Eng. Lett..

[165] Adam T., Gopinath S.C. (2022). Nanosensors: Recent perspectives on attainments and future promise of downstream applications. Process. Biochem..

[166] Ankireddy S.R., Kim J. (2019). Status and recent developments in analytical methods for the detection of foodborne microorganisms. Rec. Dev. Appl. Microbiol. Biochem..

[167] Phafat B., Bhattacharya S. (2023). Quantum Dots as Theranostic Agents: Recent Advancements, Surface Modifications & Future Applications. Mini Rev. Med. Chem..

[168] Masud M.K., Na J., Younus M., Hossain M.S.A., Bando Y., Shiddiky M.J., Yamauchi Y. (2019). Superparamagnetic nanoarchitectures for disease-specific biomarker detection. Chem. Soc. Rev..

[169] Kerry R.G., Ukhurebor K.E., Kumari S., Maurya G.K., Patra S., Panigrahi B., Majhi S., Rout J.R., del Pilar Rodriguez-Torres M., Das G. (2021). A comprehensive review on the applications of nano-biosensor-based approaches for non-communicable and communicable disease detection. Biomater. Sci..

[170] Gattani A., Mandal S., Khan M.H., Jain A., Ceaser D., Mishra A., Tiwari S.P. (2023). Novel Electrochemical biosensing for detection of neglected tropical parasites of animal origin: Recent Advances. Electroanalysis.

[171] Srivastava A.K., Upadhyay S.S., Rawool C.R., Punde N.S., Rajpurohit A.S. (2019). Voltammetric techniques for the analysis of drugs using nanomaterials based chemically modified electrodes. Curr. Anal. Chem..

[172] Chakraborty U., Kaur G., Chaudhary G.R. (2021). Development of environmental nanosensors for detection monitoring and assessment. New Front. Nanomater. Environ. Sci..

[173] Bergquist R., Lustigman S. (2010). Control of Important Helminthic Infections: VaccineDevelopment as Part of the Solution. Adv. Parasitol..

[174] Elmoghazy W., Alqahtani J., Kim S.W., Sulieman I., Elaffandi A., Khalaf H. (2023). Comparative analysis of surgical management approaches for hydatid liver cysts: Conventional vs. minimally invasive techniques. Langenbeck’s Arch. Surg..

[175] Manterola C., Rivadeneira J., Pogue S.D., Rojas C. (2023). Morphology of *Echinococcus granulosus* Protoscolex. Int. J. Morphol..

[176] Aminu N., Bello I., Umar N.M., Tanko N., Aminu A., Audu M.M. (2020). The influence of nanoparticulate drug delivery systems in drug therapy. J. Drug Deliv. Sci. Technol..

[177] Majumder J., Taratula O., Minko T. (2019). Nanocarrier-based systems for targeted and site specific therapeutic delivery. Adv. Drug. Deliv. Rev..

[178] Geramizadeh B. (2017). Isolated peritoneal, mesenteric, and omental hydatid cyst: A clinicopathologic narrative review. Iran. J. Med. Sci..

[179] Momčilović S., Cantacessi C., Arsić-Arsenijević V., Otranto D., Tasić-Otašević S. (2019). Rapid diagnosis of parasitic diseases: Current scenario and future needs. Clin. Microbiol. Infect..

[180] AlGabbani Q. (2023). Nanotechnology: A promising strategy for the control of parasitic infections. Exp. Parasitol..

[181] Aljanabi A.A., Al-Mussawi K.A., Bashi A.M. (2021). Scolicidal effects of silver-copper (core-shell) nanoparticles against *Echinococcus granulosus* protoscolices in vitro. Ann. Roman. Soc. Cell Biol..

[182] Cheraghipour K., Azarhazine M., Zivdari M., Beiranvand M., Shakib P., Rashidipour M., Mardanshah O., Mohaghegh M.A., Marzban A. (2023). Evaluation of scolicidal potential of salicylate coated zinc nanoparticles against *Echinococcus granulosus* protoscoleces. Exp. Parasitol..

[183] Look M., Bandyopadhyay A., Blum J.S., Fahmy T.M. (2010). Application of nanotechnologies for improved immune response against infectious diseases in the developing world. Adv. Drug. Deliv. Rev..

[184] Shakibaie M., Khalaf A.K., Rashidipour M., Mahmoudvand H. (2022). Effects of green synthesized zinc nanoparticles alone and along with albendazole against hydatid cyst protoscoleces. Ann. Med. Surg..

[185] Muraleedharan K., Chhabra M. (2018). Nanotechnology Applications and Potential in Parasitology: An Overview. Vet. Immunol. Biotechnol..

[186] Xu X., Qian X., Gao C., Pang Y., Zhou H., Zhu L., Wang Z., Pang M., Wu D., Yu W. (2022). Advances in the pharmacological treatment of hepatic alveolar echinococcosis: From laboratory to clinic. Front. Microbiol..

[187] Nikam P.B., Salunkhe J.D., Minkina T., Rajput V.D., Kim B.S., Patil S.V. (2022). A review on green synthesis and recent applications of red nano Selenium. Results Chem..

[188] Şenel S., Yüksel S. (2020). Chitosan-based particulate systems for drug and vaccine delivery in the treatment and prevention of neglected tropical diseases. Drug. Deliv. Transl. Res..

[189] Olawoyin R. (2018). Nanotechnology: The future of fire safety. Saf. Sci..

[190] Abdussalam-Mohammed W. (2019). Review of therapeutic applications of nanotechnology in medicine field and its side effects. J. Chem. Rev..

